# Tackling child malnutrition in Jamaica, 1962–2020

**DOI:** 10.1057/s41599-020-00536-5

**Published:** 2020-07-30

**Authors:** Henrice Altink

**Affiliations:** 1 University of York, York, UK

## Abstract

On the eve of independence in 1962, malnutrition was the largest single cause of death in Jamaica for children under one. Although child malnutrition rates have rapidly declined since 1962, today Jamaica experiences a double burden of malnutrition: the coexistence of pockets of high child malnutrition with rising levels of childhood obesity. Based on a wide range of sources, including public documents, newspaper reports, scientific studies and reports by international agencies, this article examines a gradual decline in child malnutrition and the rise of the double burden of malnutrition in Jamaica from independence to the present. It will first of all show that changes in the global economy and overseas loans and aid both aided and limited the ability of the Jamaican government to lower child malnutrition levels and also contributed to a rise in childhood obesity. Second, it will illustrate that a traditional deficit-led approach to child malnutrition was followed in post-independent Jamaica, focussing on the public and individuals as targets for intervention and using quantitative measures to trace progress. And third, it will question whether the double burden of child malnutrition will give rise to ‘healthy publics’—‘dynamic collectives of people, ideas and environments that can enable health and well-being’.

## Introduction

On the eve of independence in 1962, malnutrition was the largest single cause of death in Jamaica for children under one (Ashworth and Waterlow, 1974, p. 6). Although child malnutrition rates have rapidly declined in the island since 1962, today pockets of high child malnutrition exist alongside rising levels of childhood obesity. A recent study of 3–5-year-old children in rural Jamaica, for instance, has found that 16 per cent were nutritionally at risk: 9 per cent were moderately to severely undernourished and 6.9 per cent were overweight (Cory et al., 2014). In other words, Jamaica like so many other countries in the Global South is experiencing a ‘double burden of malnutrition’; that is, the coexistence of conventionally understood malnutrition —undernutrition due to insufficient intake of energy and other nutrients—and obesity.

This study examines the gradual decline in child malnutrition and the emergence of the ‘double burden of malnutrition’ in Jamaica from independence in 1962 to the present. Child malnutrition refers here to undernutrition and includes: stunting, low height for age; wasting, low weight for height; and underweight, low weight for age. During the period under consideration, a range of measuring methods and classification systems were used to assess child malnutrition. While the World Health Organization (WHO) and other organisations now use the term child malnutrition for children under the age of 5, studies on child malnutrition in Jamaica after 1962 measured a range of age groups, including children enrolled in primary school. Also some key interventions adopted focussed on older children, including school feeding programmes. For that reason, this study focusses not just on children under 5 but also older children.

The Government of Jamaica (GoJ), with the support of international donors, aid agencies and others, tried in various ways to lower child malnutrition rates as it realised that high levels posed a threat to the future of the island because children who suffer from malnutrition are in a critical stage of growth and if they do not catch up, are more likely not to do well in school and hence can be trapped in a cycle of poverty. And today, it undertakes efforts to lower childhood obesity rates because obese children run the risk of developing non-communicable diseases (NCDs) later in life, which in turn will place large burdens on the public health system, economy, and society. The following will show that changes in the global economy and conditions imposed by international lenders significantly affected the GoJ’s ability to lower child malnutrition levels and also contributed to a rise in childhood obesity. But it will also highlight that without international loans and overseas aid, the island would not have witnessed a gradual decline in child malnutrition in the decades following independence. In other words, it will argue that external factors, in complex interplay with local factors, acted as a double-edged sword.

This study is based on a range of primary sources, including scientific studies, newspaper reports, public documents, and reports by international donors,^1^ and is divided into five sections. The first provides historical background and shows that it was not until the interwar years before child malnutrition became a problem in Jamaica and surveys were undertaken to measure it. It also sets out the historical factors that gave rise to this problem and attempts made from the Second World War onwards to solve it. The second section zooms in on the years immediately leading up to and following independence and illustrates that methods pioneered in developed countries were increasingly replaced by interventions deemed more realistic within the local context, and that various international organisations, including the United States Agency for International Development (USAID) and the WHO’s regional office in the Americas—the Pan-American Health Organisation (PAHO)—offered crucial help that supplemented local efforts. In the 1970s, some of this aid declined at a time when Jamaica witnessed a nutrition crisis. The third section explains why child malnutrition levels did not increase during these challenging years, while the fourth examines how the GoJ tried to further lower child malnutrition in the 1980s and 1990s, amidst conditionalities imposed by international lenders that significantly affected household food security. And the last section traces the rise of the double burden of child malnutrition and mentions some methods adopted to address it.

This article will show that throughout the post-independence period, a mostly traditional deficit-led approach to child malnutrition was adopted in Jamaica; that is, focussing on the public and individuals as targets for intervention and using quantitative measures to trace progress. For instance, the GoJ ran campaigns to increase exclusive breastfeeding, set up nutrition clinics that monitored the weight of children, handed out supplementary foods for pregnant and nursing women and undernourished children, and employed public health workers who taught women how to prepare nutritious weaning foods. The conclusion will question whether it is possible for Jamaica to create what Hinchliffe et al. (2018, p. 2) have called ‘healthy publics’—‘dynamic collectives of people, ideas and environments that can enable health and well-being’—to address the double burden of child malnutrition in a context of substantial global inequalities and structural adjustment.

By examining the process by which child malnutrition rates declined and a double burden of malnutrition emerged in Jamaica after 1962, this article will contribute to several (overlapping) sets of scholarship. Firstly, it will add to the slowly growing body of work on health and medicine in the Caribbean. Despite its medical and cultural importance, nutrition has often been a marginal topic in Caribbean historiography, being only briefly addressed in discussions of diseases directly or indirectly linked to diet, such as tuberculosis or hookworm (e.g. Hoefte, 2014; McCollin, 2009). In centring upon nutrition in this way, the article will not only explicate the intricate connections between health, nutrition and political economy, but it will also shed more light on the history of post-independence Jamaica, another area that has received scant attention from Caribbean historians. Particularly, it will convey the island’s shift towards neoliberalism.

Thirdly, this study will contribute to scholarship on the history of nutrition in colonial and post-colonial contexts more generally. This body of work has focussed mostly on the first half of the 20th century. Various studies have examined attempts by colonial governments to alleviate hunger in the years before the Second World War and the science underpinning them, focussing particularly on African colonies (e.g. Brantley, 2002; Sparks, 2017). And some scholars have also used anthropometric data to assess the nutritional status of populations in order to make claims about living standards, especially in colonial contexts, or have studied nutrition as part of campaigns to improve child welfare (e.g. Austin et al., 2012; Pacino, 2015). Only more recently have scholars (e.g. Nott, 2016, 2018; Pernet and Forclaz, 2018; Redfield, 2012; Scott-Smith, 2013, 2014a, 2014b, 2015; Tappan, 2017) begun to examine approaches taken by post-independence governments and aid agencies to nutrition, focussing particularly on child malnutrition.

Most of this recent body of work focusses on Africa and is particularly concerned with the role of nutritional science and humanitarian actors in approaches to and discussions around child malnutrition. This case study, which pays less attention to nutritional science, will show that approaches to child malnutrition in Jamaica mirrored in many ways those adopted in former African colonies, including the reliance on overseas aid agencies and international lenders. Yet during the period under consideration, Jamaica never experienced (the threat of) famine and because it was more urbanised and a Small Island Developing State (SID), making it more reliant on global trade networks, it experienced the ‘nutrition transition’—a shift towards consuming more energy-dense processed foods and becoming less active, resulting in obesity and obesity-related NCDs—earlier than many former African colonies so that by the turn of the 21st century already a significant proportion of children were overweight or obese. Although now becoming more prevalent amongst children, in most former African colonies today obesity is still largely an adult phenomenon (Masters et al., 2018). This case study of child malnutrition in post-independence Jamaica, then, provides a useful addition to existing work on the history of nutrition in postcolonial contexts.

## Historical background

Child malnutrition was studied and problematised long before Jamaica gained independence and was part of broader regional and imperial-wide investigations into nutrition. For instance, in 1937 a local nutrition committee was set up that measured the nutritional status of the population, which fed into the report of the Committee on Nutrition in the Colonial Empire (Worboys, 1988; Arnold, 1994). This committee concluded that malnutrition affected a large proportion of the population, especially children as parents struggled to buy food, and that the majority of calories consumed came from bulky starches (Eisner, 1961, p. 345). Further impetus for the study of child malnutrition came in 1944 when the Colonial Office ordered an investigation into child malnutrition in the Caribbean carried out by B.S. Platt, the head of the British Medical Research Council’s Nutrition Unit. This survey concluded that the most severe cases were of children of unmarried, working mothers who struggled to breastfeed their infants and once weaned gave them a diet high in carbohydrates. It recommended, amongst others, the use of fortified flour and increased consumption of skimmed dried milk (Riley, 2005, pp. 77–88). On the basis of these various surveys, methods were adopted by the government and local charities to improve the nutritional status of children, including the provision of school lunches and an education nutrition programme (Riley, 2005, p. 78, p. 126). But these only went so far because even though it was agreed that child malnutrition was a disease of poverty, no consideration was given to the historical and structural factors that accounted for this poverty.

Like other plantation economies in the Caribbean, Jamaica had long been reliant on the importation of large quantities of food, a dependency that dated back to the formation of sugar plantations in the 17th century. Largely as a means to reduce costs, sugar planters reserved some land on their estates for slaves to cultivate their own food and supplemented their slaves’ diet with imported foods, such as salted, dried fish. The land allocated to slaves was usually the least fertile land, and slaves were only able to grow staple foods, such as yams, the surplus of which they were allowed to sell on slave markets. Thus during slavery, as Sidney Mintz (2010, p. 149) has argued, a system was set in place whereby food was divided into ‘for export’ and ‘to eat’.

These arrangements continued to structure food production and consumption in Jamaica after emancipation in 1838. Despite many ex-slaves seeking to become farmers, they struggled to obtain land. Various government schemes were created to facilitate an increase in peasant ownership and cultivation by the end of the nineteenth century, but most purchases under these programmes were small-scale (under 10 acres) and encompassed poor quality, hard to reach land (Holt, 1992, pp. 338–339). As such, though some peasants were able to procure sufficient land to cultivate bananas, coffee, and other export crops, this form of production was dominated by big landowners able to purchase the best quality land. The result was that in the decades leading up to the First World War most agricultural output was for export: between 1890 and 1910, the ratio of exports to subsistence food (e.g. yams and plantains) in total agricultural output rose from 64 to 81 per cent (Eisner, 1961, p. 170). This made the island increasingly reliant on imported foodstuffs. In 1930, the output of subsistence food totalled £4961 but the island spent £2292 on imported food, drink, and tobacco (Eisner, 1961, pp. 98–99). Also as in other parts of the Caribbean, the legacy of slavery meant that many Jamaicans attributed low status to farming and hence food ‘to eat’ but high status to imported food (Wilson and McLennan, 2019, p. 174).

Changes in demographic and economic circumstances made it even more difficult to secure adequate nutrition after the First World War. Between 1921 and 1943, the population increased by 1.6 per cent per annum (Eisner, 1961, p. 135) but the availability of food for domestic consumption did not rise accordingly. And food security worsened during the world-wide economic depression when the price for export products sharply dropped and the bill for imported food rapidly increased, while wages declined and unemployment rose. In the 1930s, then, many parents struggled to feed their children sufficient and nutritious food, especially those in urban areas who lacked domestic gardens. During the Second World War, the cultivation of food for domestic consumption was increased as a result of various government schemes so that the production of corn, peas, and beans increased substantially and the island was able to supply about 75 per cent of its condensed milk and meat. Also the local use of bananas previously exported to the UK and North America led to a decline in demand for imported food (Füllberg-Stolberg, 2004, pp. 115–116). But after the War, the importance of food crops for local consumption was quickly forgotten, even though population levels continued to rise and, as the following section will show, research showed that many children suffered from protein deficiency.

## Measuring and understanding child malnutrition

In the 1950s, child malnutrition in Jamaica became defined as a medical-nutritional problem. Although local health researchers, centring around the Tropical Metabolism Research Unit (TMRU) at the University College of the West Indies, agreed that child malnutrition was largely the result of poverty, they did not assess the extent to which household income and other socio-economic factors contributed to child malnutrition (Waterlow, 1992). Instead, they measured the degree and nature of child malnutrition based on existing statistical medical data, such as infant mortality rates, and some experimental research. They were also more concerned with treatment of severe cases, especially of extreme protein deficiency, than with prevention (e.g. Waterlow and Wills, 1960). In other tropical British colonies, there was a similar focus on protein deficiency which, as Nott (2018) has argued, was a continuation of the work of the Committee on Nutrition in the Colonial Empire that had presented child malnutrition as a problem of quality not quantity. Only gradually was a more complex understanding of child malnutrition advanced, leading to the adoption of the now widely used protein-energy malnutrition (PEM) spectrum, which covers a range of conditions from a consistent lack of dietary protein and/or energy.

The GoJ and various UN agencies operating in the island in the 1950s equally medicalised child malnutrition. The GoJ adopted various schemes largely based on child feeding practices used in developed countries, such as cod liver oil distribution schemes (Cook and Yang, 1974, p. 133), while UNICEF, for instance, sponsored a free milk and school feeding programme. Jamaica was not unique in this regard. Tappan (2017), for example, has shown how through the distribution of baby formula and other schemes child malnutrition became medicalised in Uganda in the 1950s. By the late 1950s, however, nutritional researchers increasingly began to question such approaches and started to favour the vernacularisation of nutritional advice (Nott, 2016, p. 239); that is, adapting nutritional science to local contexts, paying attention not just to people’s living conditions but also their cultural beliefs and values.

The WHO, for instance, questioned the suitability of cod liver oil distribution schemes in tropical regions where sunlight exposure throughout the year allowed for optimal levels of Vitamin D, and started issuing guidelines for large-scale nutrition surveys that included attention to social and cultural factors (Jelliffe, 1955).

Informed by WHO guidelines, at least seven large-scale anthropometric surveys—measuring and weighing children— were undertaken in British Caribbean (former) colonies in the 1960s. The use of nutrition anthropometry in the colonies was not without its problems. The use of the so-called Gomez scale,^2^ for instance, reinforced a racialised view of the ‘normal’ and ‘abnormal’; it divided malnutrition into mild, moderate and severe forms, but used children in Boston, measured between 1930 and 1956 and of mostly European descent, for its reference population (Scott-Smith, 2013, pp. 922–923; Gueri et al., 1980, p. 774). Nevertheless, defining child malnutrition in relation to both protein and calories, these surveys concluded that there was little severe malnutrition in the British Caribbean but that about 30 per cent of children suffered from mild to moderate malnutrition (Jelliffe, 1971a, 1971b p. 146). And to further illustrate the shift towards vernacularisation and a more multi-causal, mixed-methods approach, by the early 1960s anthropologists had joined medical scientists in researching child malnutrition, using interviews and other tools to understand the social, cultural and economic factors affecting the growth of Jamaican children.

The first survey undertaken in Jamaica after independence was carried out in 1963 by the nutrition unit of the Jamaican Scientific Research Council. It looked at the food intake of 665 pre-school children from 369 families in 59 different areas. Another survey took place in 1970, carried out with support from the Caribbean Food and Nutrition Institution (CFNI), a specialist centre of PAHO set up in 1967. And a PAHO study into nutritional problems in the Caribbean from 1963 to 1964 also included Jamaica (Aykroyd, 1965). These various studies tried to estimate the extent to which malnutrition was the major cause of death for young children in order to highlight the severity of the issue and establish the daily food intake of young children in order to recommend specific interventions (Israel, 1984). Waterlow and Ashworth (1974, p. 7, p. 12), for instance, concluded that malnutrition and gastroenteritis accounted for 40–60 per cent of deaths of Jamaican children aged 1–24 months, and that 90 per cent of children under 1 received less than the recommended daily calorie and 85 per cent less than the recommended protein intake.

The various Jamaican nutrition studies concluded that the high rate of infant deaths caused by malnutrition was the result of various socio-economic and cultural factors, most notably poverty, mirroring similar findings in other British (former) colonies (Nott, 2016, p. 239). Although Jamaica witnessed economic growth in the 1960s, poverty levels were high. Unemployment increased from 13.5 to 17 per cent between 1960 and 1969. And while per capita national income increased by 4.1 per cent between 1950 and 1968, income distribution was so skewed that only 30 per cent of the population enjoyed rising incomes (Jefferson, 1972, p. 52). In fact, the weekly income of the poorest 30 per cent fell by 24 per cent between 1958 and 1968 (Girvan et al., 1980, p. 115). Furthermore, the price of food increased by some 32 per cent in the 1960s, partly caused by an increase in import prices following devaluation (Jefferson, 1972, p. 72). As about 50 per cent of the income of lower-income households was spent on food, this increase in food prices significantly affected the amount and quality of food that they could give their children. That child malnutrition in the 1960s was highest in single female-headed households exemplifies the link between poverty and child malnutrition. These households were generally poorer than two-parent households as few men gave the unmarried mothers of their children financial support (Desai et al., 1969, p. 311) and most of these women undertook low-paid work. As Platt had already observed in the 1940s, these women as a result rarely breastfed for more than 3 months which further put their infants at risk of malnutrition.

Early and exclusive breastfeeding has long been seen as a means to enhance the survival chances of infants; breastmilk is easily digested and uncontaminated with infectious organisms so it protects against gastroenteritis. The various studies carried out in Jamaica in the 1960s found a rapid decline in breastfeeding: few mothers breastfed beyond 3 months and the trend was towards less breastfeeding and earlier introduction of the bottle (see Table 1). Social and economic pressures largely explain this change. Many lower-class mothers introduced the bottle to imitate those above them because the better-off tended to only bottle feed. But by the late 1960s, as in various other developing countries (see Nott, 2018, p. 779; Tappan, 2017) these women were also increasingly targeted by commercial milk companies. Newspaper adverts, billboards and other advertising hailed formula as ‘the best food for infants’. So-called ‘milk nurses’ working for commercial milk firms obtained names of women who had recently given birth in the hospital and then visited them at home and gave them samples. Some hospital staff also readily gave women who struggled to breastfeed the bottle. Under such pressure, many Jamaican women were convinced that expensive baby formula was best (Grantham-McGregor and Back, 1970; Reddy, 1971).

Like women in other tropical countries, Jamaican women found it difficult to sterilise bottles and teats (many only had one bottle and not a separate pan to sterilise it) and because formula was expensive, they also tried to stretch it so that the baby was given diluted milk. One study estimated that it would cost half a family’s weekly income to feed a child formula according to the guidelines on the tin (Jellife, 1971, p. 182). Mothers who could not afford to buy expensive formula often used condensed milk, which was cheap but high in sugar. By the late 1960s, more women started to use dried skimmed milk, which contained more protein than whole milk and was cheaper (Aykroyd, 1965, p. 146).

Jamaican mothers’ assumptions about appropriate weaning foods was also singled out in various studies as a factor accounting for high child malnutrition rates. For example, mothers rarely gave children under two fish, meat or sweet potatoes as they thought that these foods produced worms. The high status attached to commercially produced weaning foods also meant that they forewent bananas and other local foods for weaning mixtures (Aykroyd, 1965, p. 146; Ashworth and Waterlow, 1974, p. 29). A study undertaken by Arlene Fonaroff (1968), a visiting research fellow at CFNI, in the late 1960s, found that even many mothers who attended child welfare clinics held on to traditional, non-nutritious weaning practices. And Waterlow and Ashworth (1974) also singled out the gendered distribution of food in the household as a contributing factor of child malnutrition: the male head received most of the meat at mealtimes, while toddlers had to content with high-starch, semi-solid paps. Yet these studies did not dismiss this as evidence of ‘superstition’ and ‘backwardness’, like studies into child malnutrition during the colonial period had done in order to avoid addressing structural causes. Nor did they advocate instruction in ‘proper’, i.e. Western, child feeding methods. Rather, solutions were offered that actively worked with local beliefs and values and considered the context in which Jamaican mothers prepared food.

It was strongly recommended that infant feeding was taught through practical demonstrations, using foods, cooking equipment and methods that were feasible in local homes (Ashworth and Waterlow, 1974). Such teaching had to discourage the use of non-nutritious food stuffs, such as white potato (possessing a high water content), and to persuade mothers to abandon harmful practices, including withholding food from children when ill. Yet mothers were not to be dissuaded from traditional practices that were neither harmful nor beneficial, such as giving children non-toxic ‘bush teas’. In fact, medical staff were encouraged to actively work with traditional beliefs. For example, they were told that they should work with the belief in duppies— ghosts or spirits—telling mothers that their children would be less susceptible to duppies if fed an adequate diet (Fonaroff, 1968; Aykroyd, 1965).

Gradually, the teaching of infant feeding did become more ‘realistic’, ‘affordable’ and ‘local’ as it did in many other developing countries, such as Uganda (Tappan, 2017). PAHO (1970), for instance, drew up feeding guidelines for the Caribbean but left out ‘those specific details that require modification to suit local circumstances’. And slowly attempts were also made to implement various proposals to prevent a further shift towards bottle feeding (De Morales and Larkin, 1972; Ashworth and Waterlow, 1974). For instance, in 1974 milk nurses were banned from the Victoria (maternity) Jubilee hospital and the hospital of the University of the West Indies (Gleaner, 7 April 1974).

Recognising that poverty was a main cause of child malnutrition, it was also recommended that more and better day-care facilities be set up for children of working mothers (Ashworth and Waterlow, 1974, p. 79). And even more commonly recommended was an increase in maternal and child welfare clinics. By the late 1960s, there was a shortage of district midwives and public health nurses so that these clinics were often held only every other month and they only reached about one-third of all infants born (Ashworth and Waterlow, 1974, p. 75). From the late 1960s onwards, the number of clinics rapidly increased, providing easy access to clinics that monitored the weight of children and offered supplementary foods to pregnant and nursing women and at-risk children, where needed.

State interventions in this period were also stimulated by international agencies and encompassed a mix of technical and structural solutions. For instance, PAHO (1970) and other aid agencies active in the region placed significant responsibility on Caribbean governments, asking them for instance to fortify all imported dried skimmed milk with vitamin A; distribute low-cost iron and folic acid supplements for children under 2 years through health clinics; and subsidise foods for children, such as multi-mix weaning foods made from locally available ingredients. In line with this advice, in 1967 the GoJ assumed responsibility for the USAID-sponsored Food-for-Peace programme, which included a school feeding programme and the distribution of dried skimmed milk and CSM (a mixture of cornmeal, soya and powdered milk) to nursing and pregnant women and children up to the age of 2 years. The inclusion of CSM limited somewhat the drive towards localisation as it built on both the colonial interest in protein deficiency and the high modernist mentality to replace tradition with scientific and technological interventions (Scott-Smith, 2015).

However, by the late 1960s, the Food-for-Peace programme only reached a small proportion of at-risk children because their mothers tended to be poorer and less educated and therefore less likely to attend the child welfare clinics that distributed the milk and CSM (Ashworth and Waterlow, 1974, p. 31).^3^ Expanding its efforts, the GoJ began to control the price of some 15 food items to protect low-income groups, pursued a policy of import controls to encourage the use and production of local foods stuffs, ordered research into ‘indigenous agricultural produce’ that could be marketed locally, and used radio to convey messages about healthy child nutrition (Ashworth and Waterlow, 1974, p. 24, p. 39; Ministry of Trade and Industry, 1970).^4^ It was not unique in doing so. For example, Ghana, which had gained independence 5 years earlier, equally adopted policies to reduce imports and lower food prices (Robins, 2018).

The various measures adopted by the GoJ in the late 1960s and early 1970s, then, illustrate as Mozaffarian et al. (2018, p. 3) have argued, the extent to which public health nutritionists in developing countries in the 1960s embraced a policy that focussed on increasing calories and selected micronutrients through producing low cost, energy dense staples accompanied by the fortification of staple foods and food assistance programmes for vulnerable populations. Although during this period, the focus shifted from treatment to prevention and more attention was paid to local context and culture, measures adopted did little to address one of the main causes of child malnutrition: household poverty. The following section will demonstrate that it took a new government to offer a more holistic approach to the problem of child malnutrition, which combined a reliance on global agencies for food and loans with the use of novel health coalitions.

### Pioneering new methods

The 1972 general election led to a People’s National Party (PNP) government led by Michael Manley, which adopted a democratic socialist programme that included policies to reduce poverty and social inequalities. As suggested by various UN agencies, a year after it took office, it adopted a nutrition programme that addressed nutrition from different angles—health, agriculture, and education—and focussed on food supply, food demand, and the ‘biological use of food’ (e.g. mother and child health programmes) (WHO, 2008, p. 147; Cook and Yang, 1974, pp. 134–136). The Manley government also devised a national food and nutrition policy that aimed to achieve by 1980 adequate nutrition and dietary well-being for all; more locally produced foods; and the elimination of malnutrition in vulnerable groups, including young children (Gleaner, 5 August 1976).

The import-substitution scheme Growing and Reaping our Wealth (GROW), which was launched in 1973, was mostly a response to a rising bill of imported food stuffs, caused largely by devaluation. The food bill increased from Jamaican dollars (J$) 43.3 million in 1968 to J$60.2 million in 1971 and rose even further after the oil crisis (Ministry of Agriculture, 1973). In fact, between mid-1973 and mid-1975, the food price index increased by 90 per cent: in the space of 20 months food prices had nearly doubled so that half the population spent at least 60 per cent of their income on food. The rise in wages until 1975 counteracted this price rise to some extent but unemployment levels also soared from 22.9 per cent in 1972 to 26.8 per cent in 1980. Furthermore, the flow of food aid supplied by USAID, including food supplements for pregnant and nursing mothers and infants dropped sharply, as the US turned against Jamaica because of the PNP government’s attitude to ‘foreign investors, to trade policy, foreign loans, aid alliances with other Third World countries, and matters of formal diplomacy’ (Kaufman, 1985, p. 87). As a result, in the mid-1970s, Jamaica experienced a nutrition crisis. This along with financial and other constraints limited the GoJ’s ability to meet the aims of its national food and nutrition policy (Marchione, 1977, pp. 62–64).

Considering the economic decline, which was unprecedented in post-war Jamaica, it is remarkable that child malnutrition did not increase during the 1970s (see Table 2). Headline figures, however, mask pockets of high child malnutrition, which became particularly pronounced in certain rural areas as the decade progressed. Several factors explain why there was an *overall* improvement in the nutritional status of young children during the 1970s. First, a rapid increase in the informal sector—jobs not recognised as normal income and on which no taxes are paid, including illegal activities—provided unemployed or underemployed communities with some income. Second, policies adopted by the PNP government led to better income and employment, especially for rural households. Particularly important here was Project Land Lease under which privately held lands were leased by the government, and then (for period of 5 years) to small farmers in the area to allow them to supplement their own holdings. By 1980, almost 38,000 farmers had been placed on 75,000 acres of land (Stephens and Stephens, 1986, p. 74).

Third, the PNP government expanded existing food subsidies and school feeding programmes (Riley, 2005, p. 177). In December 1971, the Jamaica Labour Party (JLP) government had signed a Food-for-Peace agreement with USAID for a new school feeding programme. A government-owned company Nutrition Production Limited (NPL) was set up, which supplied children in the parish of Kingston and St. Andrew with a bun (and initially also a patty—a pastry with various fillings, such as beef or chicken, inside a flaked shell) and a half pint of milk. This protein-enriched meal, for which children only paid a few J$ cents as the scheme was heavily subsidised, provided one-third of the required daily calorie and protein intake. The milk, for instance, was a re-combined milk consisting of skimmed milk powder fortified with butter, oil or soya and flavoured with vanilla. It was largely because most schools in the parish did not have kitchens that the programme opted for conveniently packed buns or patties (Gleaner, 17 December 1971, 6 October 1975, 17 March 1978). Outside Kingston and St. Andrew, schools under the Food-for-Peace programme were given oil and other supplies and funds to buy meat and vegetables to provide children with a cooked lunch. But when NPL facilities were increased, gradually schools in other parts of the island could also opt for what became known as the ‘nutribun scheme’.

Nutribuns were developed by USAID with a recipe designed around a ‘base’ product that could be modified using any number of locally available substances and were first used in the Philippines in 1970 following a series of typhoons (USAID, 1972). This flexibility was central to what Scott-Smith (2015, p. 245) has called ‘ready-made solutions in humanitarian action’, and by 1978, some 120,000 school children in Kingston and St. Andrew—and 100,000 in rural areas—were supplied with nutribuns or a cooked lunch (Gleaner, 10 September 1978).^5^ Initially this school feeding programme had a positive impact on the nutritional status of schoolchildren. However, effects diminished over time, largely because children were not obliged to eat the food, and once the novelty had worn off they opted for cheap but less nutritious snacks sold at the school gates (Edwards, 1984, p. 79).

And a fourth factor why overall child malnutrition levels did not increase in the 1970s is the PNP government’s emphasis on primary healthcare, the first line in the prevention and treatment of child malnutrition. From the mid-1970s till the early 1980s, Jamaica had a thriving system of primary healthcare. In the early 1960s, there were only 73 health centres but by 1977 there were already 382. In 1978, the Ministry of Health decided to build 430 new health centres with loans from international donors. About 365 of these were eventually built so that most people were able to access a clinic within 10 miles of their home (Riley, 2005, pp. 179–180).

Many rural clinics employed officers called Community Health Aides (CHAs). This use of lay health workers first began in 1969 when the GoJ, the University of the West Indies and Cornell University Medical College set up a rural health project in Elderslie, in the parish of St. Elisabeth, which had a high rate of infant mortality caused by malnutrition (Alderman et al., 1973, p. 1168). The Manley government made CHAs an integral part of its expanded primary care system, largely driven by a shortage of trained medical staff.^6^ By the end of the 1970s, some 1300 CHAs were employed, each serving about 600 people. CHAs did a lot of routine clinic health work but focussed mainly on maternal and child welfare. They had to encourage exclusive breast feeding; improve weaning practices; encourage a more balanced and affordable family diet by reducing the reliance on imported foods and increase the use of home-grown alternatives; and increase regular attendance at clinics so that children’s weight could be monitored and at-risk cases be given supplements (Riley, 2005, p. 180; Marchione, 1984). This model has since been copied by many other countries.^7^

The impact of the increased use of CHAs on malnutrition varied from parish to parish. In Elderslie, for instance, it did not reduce overall levels of malnutrition but succeeded in lowering high mortality associated with nutritional deficiency because children that were mildly or moderately malnourished were sought out and then treated in their homes. Yet in Hanover parish, malnutrition levels of children under 2 fell by about 40 per cent after the introduction of CHAs. This was largely because the parish employed nearly twice as many CHAs as other parishes and also had a relatively continuous supply of food aid from the US (Marchione, 1984, pp. 229–234).

From the mid-1970s onwards, the GoJ had to scale back various policies that benefited the nutritional standard of the poor. For example, under agreements with agencies like the IMF, the price controls on certain food items were repealed and CHAs were no longer able to hand out supplementary food. The first IMF agreement was signed in 1977 and more followed, each with their own set of conditions that aimed largely to reduce government expenditure including spending on healthcare. The second half of the 1970s also witnessed more unemployment, a further hike in food prices, and food shortages. The latter two impacted the school feeding programme, reducing the nutritional value of the nutribun (Gleaner, 22 August 1980). Nevertheless, the government continued to implement its national food and nutrition policy. In 1978, for instance, it embarked on a nutrition education programme, which began with a mass media campaign that aimed to convey five basic messages, including that babies should be exclusively breastfed for the first few weeks and then weaned onto nutritious food (Gleaner, 14 February 1978). Furthermore, the Jamaican Scientific Research Council expanded its import-substitution programme, focussing on such things as using indigenous roots and tubers for composite flour formulations (Gleaner, 30 March 1978). This along with an increase in self-sufficiency amidst high food prices led to a 10–20 per cent increase in domestic food production between 1977 and 1979, which further explains why the overall nutritional status of children did not decline in the second half of the 1970s (Weiss, 2004, p. 465).

In the 1970s, then, local and external factors combined to produce a nutrition crisis. The GoJ adopted an innovative scheme to monitor children at risk of malnourishment and also various policies to ensure the food security of low-income households. But the decline in child malnutrition during this decade also owed much to overseas food aid and loans. The loans, however, came with conditions that also posed a threat to the nutritional status of children as the following section will show.

## Neoliberalism and child malnutrition

The 1980 election, the bloodiest ever with more than 800 people dead, changed Jamaica’s political landscape: democratic socialism gave way to neoliberalism when the JLP, led by Edward Seaga, assumed government. In the first 3 years after the election, international investment and loans increased and facilitated economic growth (Danielson, 1996, p. 101). But in 1984 and 1985, the economy contracted and income from the major contributors to GDP—tourism and bauxite—began to decline. This along with the structural adjustment programmes (SAPs) imposed by the IMF and World Bank, which effectively stipulated free market programmes and a reduction in fiscal deficit (Anderson and Witter, 1994, p. 42), led to fiscal austerity. Several measures adopted to reduce the fiscal deficit directly or indirectly affected the nutritional status of children, including cuts in public employment; reduced spending on health services—from 8 per cent of total public expenditure in 1977–78 to 6 per cent in 1984–85; and the introduction of fees for many healthcare facilities (Danielson, 1996, p. 106).^8^ Furthermore, as stipulated by lenders, the Jamaican dollar was devalued and this with the removal of food subsidies led to a massive hike in food prices. A least-cost basket of food for a household of five, as devised by the Ministry of Health, increased by 67 per cent between December 1983 and July 1985 (Boyd, 1988, p. 139).

In many other developing countries, fiscal austerity programmes adopted at the insistence of international lenders led to an increase in child malnutrition, particularly because state-funded nutrition programmes were significantly cut (e.g. Nott, 2018, pp. 783–784). As Table 3 shows, in Jamaica fiscal austerity did not increase overall levels of child malnutrition. Yet the poorest parts of the island—especially rural areas—witnessed high rates. In 1989, for instance, while 30 per cent of the total Jamaican population fell below the poverty line, the figure for the rural population was 40.9 per cent (Anderson and Witter, 1994, p. 38). It was partly because there were more jobs available, wages were higher, and it was easier to supplement income by tapping into the informal labour market that urban areas witnessed lower levels of child malnutrition (Anderson and Witter, 1994, p. 33). For instance, in 1984 the average rate of moderate and severe child malnutrition in the urban parish of Kingston and St. Andrew was 3.7 per cent but in the rural parish of Westmoreland it was 9.3 per cent. Nonetheless, even within urban parishes there were areas with considerable pockets of child malnutrition— reaching as high as 10.5 per cent in some parts of Kingston and St. Andrew (Rainford, 1984, pp. 71–73).

Poverty in rural areas was largely the result of long-term factors, including the aforementioned focus on export crops, inequitable access to land, limited availability of good agricultural land, and population growth. The SAPs further increased rural poverty through the strong bias against production for the domestic market (IICA and IFAD, 1994, p. 60, p. 70), the mainstay of small farmers. Also many rural areas witnessed frequent natural hazards that impacted upon a household’s food security through a reduction in agricultural output and income and an increase in the household’s food budget. In fact, the parishes that most experienced natural hazards showed particularly high levels of child malnutrition, such as St. Catherine, St. Elisabeth and St. Thomas.

That overall levels of child malnutrition did not rise in the early 1980s was largely because in most parts of the country a primary healthcare system continued to exist, even though half of the CHAs were laid off. Also people adopted a variety of coping mechanisms: many sought recourse to the informal economy, while others used up their savings or relied on overseas remittances. The reduction in food imports and the end of food subsidies furthermore made people more self-sufficient (Omawale and McLeod, 1984; Fletcher et al., 1988).^9^ Finally, breastfeeding increased as household incomes fell and women were disproportionately affected by unemployment (female unemployment levels being at least twice that of males). In 1983, 63.2 per cent of all new mothers exclusively breastfeed for the first 6 weeks (Ministry of Health, 2014, p. 14).

New programmes compensated ineffectually for the SAP-induced cuts in expenditure. In 1984, a Food Aid Programme (FAP), financed amongst others by USAID and the UN World Food Programme to the tune of J$141 million, was adopted that consisted of two key elements. The first was a food stamp programme for most-at-risk groups, including pregnant and nursing women, all children under 5 registered at a health centre, and some moderately to severely malnourished children. The stamps entitled them to buy powdered skimmed milk, cornmeal, and rice. In 1988, some 23 per cent of all households and 38 per cent of children under 5 received food stamps. The second element was a school feeding programme under which some 600,000 children received the nutribun or a cooked lunch either for free or a small fee (Grosh, 1992, pp. 24–25). However, by the late 1980s schools often failed to receive enough buns and as NPL did not receive sufficient milk powder supplies, children were increasingly given sweetened juice instead of milk (Gleaner, 20 December 1989). As in the 1970s, children quickly grew tired of the bun and swapped it for cheap energy-dense food sold by street vendors (Gleaner, 1 July 1988).

Compared to the food subsidies of the 1970s and early 1980s, food stamps succeeded better in meeting the needs of the most at-risk groups.^10^ For example, only 34 per cent of food subsidies went to the poorest 40 per cent of the population but stamps went to 57 per cent of the poorest 40 per cent. Yet only half of households with malnourished children received food stamps, largely because of problems with registration and administration of the programme (Grosh, 1992, p. 31). Also the value of the stamps amounted to less than one-fourth of the weekly low-cost food basket for a family of five. Food stamps remained in place until 2001 when poor households were transferred onto the Programme for Advancement through Health and Education (PATH), a conditional cash transfer programme.

Food stamps and other forms of social support offered by the JLP government in the 1980s targeted primarily those who fell below the poverty line but little was done to limit the impact of the SAPs on the working non-poor, whose consumption levels, including food, declined. In 1989, some 46 per cent of workers earned less than half of the minimum family income as wages did not keep pace with inflation. This was particularly the case for female heads of households, with 54 per cent of women earning less than the minimum family income (Mullings, 2013, p. 396). They along with many others increasingly relied on churches and charities, such as Food for the Poor, that offered a variety of nutrition outreach programmes filling a gap that government was not able to fill (Gleaner, 14 November 1986). This was also common in many other developing countries. Nott (2018, p. 783), for example, has observed that the SAPs in many African countries in the 1980s increased the presence of NGOs and charities in the healthcare sector, including nutrition programmes.

The 1989 election, in which food shortages and price hikes played a role, were won by the PNP. The idealism that the PNP had displayed in the 1970s was quickly replaced by realism. Barely 3 months after the 1989 election, Prime Minister Manley negotiated another IMF loan. In 1992, P.J. Patterson took over from Manley and he continued to steer the country on a neoliberal path, which directly and indirectly affected the nutritional status of children. The devaluation of the Jamaican dollar in January 1990 led to an increase in the consumer price index for food. The low-cost basket of food items for a household of five, for instance, increased by some 220 per cent between 1995 and 1996 (Ministry of Health, 1996). In addition, there were also food shortages and a decline in spending on primary healthcare from 20.5 to 15.7 per cent of the total healthcare budget (King 2001, 38). In 1989, 6.6. per cent of children under the age of 3 who attended clinics were moderately to severely malnourished, rising to 7.4 per cent in 1992 (Irons, 1994, p. 2). Parishes in the southeast together with Trelawney in the west had the highest prevalence but inner-city areas in Kingston and St. Andrew and urbanised areas in rural parishes also had clusters of high child malnutrition. A study of some 800 children in grade 5 in 16 rural schools in 1995–96 found that 14.7 per cent suffered from anaemia and more than 10 per cent had not had any breakfast (Hutchinson, 1996, pp. i–ii).

The PNP government in the 1990s first of all tried to improve the nutritional status of children by increasing the number of food stamps for families with malnourished children and later also offered a food aid programme to supplement the diets of those on food stamps (Ministry of Health, 1996). Second, it undertook a national breastfeeding campaign to encourage exclusive breastfeeding for the first 6 weeks as recommended by the WHO but its target of 70 per cent was never achieved (Ministry of Health, 1996).^11^ Third, it extended the nutrition clinic scheme. From 1986 onwards, monthly nutrition clinics had been held at local health centres in high risk areas. The clinics monitored the growth of children under 3 and gave those malnourished a cereal-based energy supplement until they had achieved the desired weight for height. They also offered meal demonstrations and dietary counselling. In 1989, nearly 2000 children attended these monthly clinics, rising to some 5000 in 1992, again illustrating how the termination of food subsidies and rise in food prices affected child nutrition (Ministry of Health, 1995, pp. 20–22; Irons, 1994). Yet the clinics did not reach all malnourished children largely because many CHAs, who had to refer children to the clinic, were laid off (Anderson and Witter, 1994, p. 51; Irons, 1994). When several clinics ceased to exist because of staff shortages and other factors, government began to put more resource into this initiative and by 1994 there were some 77 nutrition clinics in operation which provided care for 1400 children (Ministry of Health, 1995, p. 20).^12^ To enhance food security, the GoJ also relied on various international organisations, NGOs and charities. The Food and Agricultural Organization of the United Nations (FAO), for instance, sponsored a project to set up backyard gardens in two urban communities, while Food for the Poor ran breakfast programmes for adults and children, gave fishermen in some communities equipment, ran a soup kitchen and set up chicken farms (Valstar, 1999; Gleaner, 11 November 1992).

In 1990, the UN World Food Programme became the main sponsor of the school feeding programme, supplying flour, sugar and milk powder. It was free only for the most needy children. This meant that many children were unable to buy the more nutritious option—the cooked lunch—as this cost J$6–10 compared to J$1 for the nutribun. In 1997, the World Food Programme stopped sponsoring the school feeding programme (Gleaner, 6 June 1997). NPL as a result faced dwindling stocks and heavy purchasing costs. In addition, schools that offered a cooked lunch only received supplies that could feed about a quarter of all enrolled children.

Inadequate supply and price were not the only reasons why only 20 per cent of all school children had the nutribun or the cooked lunch option. Table 4, based on a study of 16 schools in rural Jamaica, indicates that by the late 1990s most children, especially the older ones, purchased their lunch outside the school gate. The children who had snacks and sweets for lunch had a lower intake of protein and iron than those who had the nutribun, the cooked lunch, or who went home for lunch. Public health nutritionists at the time already warned about the impact of these unhealthy dietary habits as these children moved into adolescence and adulthood (Walker et al., 1998). Table 4 also shows that some children had nothing or hardly anything to eat or drink. This is partly because nutribun was associated with lower-class status and thus many children forwent it even if they could afford it (Jennings, 2016, p. 79).

**Fig. 1:**
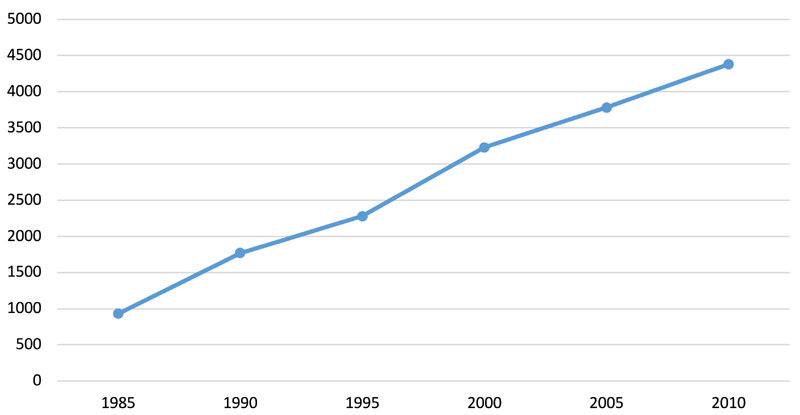
GNI per capita, 1985–2010. Reflects the rise in the total domestic and foreign value added claimed by Jamaican citizens between 1985 and 2010 expressed in international dollars using purchasing power parity. GNI per capita provides an aggregate measure of income. Note: Data sourced from the World Bank (https://data.worldbank.org/country/jamaica).

As in many other developing countries in the 1980s and 1990s, then, SAPs with their fiscal austerity measures negatively affected the food security of poorer households in Jamaica so that there were pockets of high child malnutrition. Yet they also stimulated economic growth, particularly through their emphasis on free market programmes. Figure 1 shows that in the 1980s and 1990s gross national income (GNI) increased. This largely explains why overall child malnutrition levels (Tables 3 and 5) did not massively increase during these decades. The following section will discuss another impact of the SAPs on child nutrition: rising childhood obesity levels, facilitated by trade liberalisation and a reduction in the barriers on foreign capital.

## The double burden of malnutrition

Since the late 1990s, there has been a downward trend in child malnutrition as Table 5 shows. While the child malnutrition rates in this table are low compared to other developing countries and far below levels in the late 1970s, they are still a concern because undernourishment can critically harm the physical and mental development of children.

As in previous decades, current malnutrition rates reflect pockets of poverty. Figure 1 masks gross inequalities in income distribution. Many Jamaicans have not experienced improved income levels since the early 1990s. Today poverty is particularly pronounced in rural areas. While in 2013, some 20 per cent of the urban population were poor, in rural areas it was more than 31 per cent. Also some 61 per cent of rural households today receive a cash transfer through PATH, compared to some 50 per cent in Kingston and 54 per cent in other towns (Bertelsmann Stiftung, 2018, p. 17). Yet within urban areas there are also pockets of high poverty. Poor households have struggled more than others with food price hikes, spending more of their income on food and forcing many to consume fewer calories (Myrie and Robinson, 2013). That it is mainly children whose parents depend on PATH that are underweight amply demonstrates the correlation between poverty and child malnutrition.

**Fig. 2:**
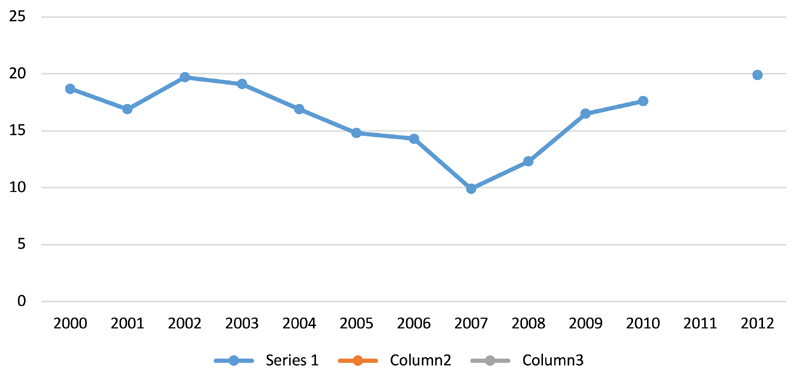
Poverty headcount ratio at national poverty line (% of population), 2000–2012. Gives an indication of the percentage of the population living below the national poverty line. National estimates are based on population-weighted subgroup estimates from household surveys. Notes: At $1.90 a day (PPP 2011). Data for 2011 missing. Data sourced from the World Bank (https://data.worldbank.org/country/jamaica).

**Fig. 3:**
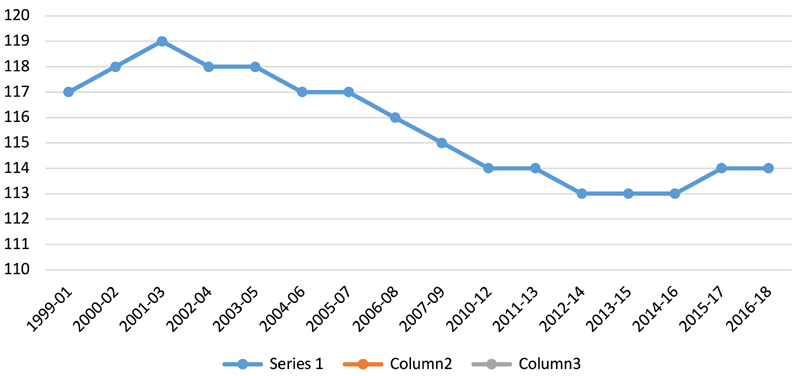
Average dietary energy supply adequacy (%), 3-year average, 1999–2018. Expresses the dietary energy supply (DES) as a percentage of the average dietary energy requirement (ADER). Jamaica’s average supply of calories for food consumption has been normalised by the average dietary energy requirement estimated for its population to provide an index of adequacy of the food supply in terms of calories. Note: Data sourced from FAOSTAT (http://www.fao.org/faostat/en/#data/FS).

Especially since the 2008 economic crisis, poverty levels have risen, as Fig. 2 indicates. They have come down since but even today more than 17 per cent of Jamaicans live below the poverty line (Gleaner, 3 August 2019). The 2008–09 global financial crisis led to increased unemployment; depreciation of the Jamaican dollar; a decline in overseas remittances; an increased debt burden; and rising food prices. For instance, the price of callaloo, a popular local leafy green vegetable, increased from J$15–20 in 2007–08 to J$60 in 2008–09 (Thomas-Hope et al., 2017, p. 27). And Fig. 3 demonstrates that the 2008–09 crisis has significantly affected food access and availability. In 2009, a new agreement was signed with the IMF, which came with more conditions to reduce fiscal deficit than previous loans. It increased certain taxes, especially the general consumption tax, raising the price of certain foods. It also imposed reduced spending on health, education and other public services, including state-run nutrition clinics and programmes (FAO, 2014; Johnston and Montecino, 2011). As a result, many schools now run breakfast clubs, sponsored by companies, churches and charities (Gleaner, 12 November 2013).

As Table 5 indicates, in the 1990s overweight and obesity levels for children started to rise and they now far exceed malnutrition levels. The neoliberal path taken by the GoJ since the 1980s to meet conditions imposed by international lenders have not only done much to increase food insecurity amongst low-income groups but also facilitated a rise in income. Between 1985 and 1993, for instance, per capita income increased by some 3 per cent per annum. Although income was not equally distributed, as mentioned, this rise did mean that many Jamaicans from the 1980s onwards were able to spend more money on non-essential items and they increasingly started to replace basic staple commodities with imported energy-dense, processed foods. As a result, about one quarter of national food consumption comes from outside Jamaica (Karfakis et al., 2011, p. 4). The reduction in trade restrictions that came with the SAPs in various ways increased the availability of imported, high-energy processed food, making it for instance easier for multinational fast f ood chains to set up branches. In 1985, for example, the first branch of Burger King was opened and today there are some 18 outlets.

Most of the new fast food chains in the 1980s and 1990s, however, were locally owned, including Juici Patties (1980), Mother’s (1981) and Island Grill (1991). A reduction in corporate tax from 45 per cent in 1986 to 33 per cent in 2006 and other fiscal incentives that the GoJ was encouraged to adopt by the international lenders go some way to explain the rise in locally owned fast-food outlets and also the growth of local food producers and distributors like Wisynco and Grace Kennedy (Trading Economics). Between 1992 and 1999, the consumption of meals outside the home increased by 9 per cent and these are mostly fat, energy-dense meals (FAO, 2010). Eating out is becoming increasingly popular amongst young Jamaicans. A study of the dietary consumption patterns of 15- to 19-year-olds in 2006, for instance, found that 57.9 per cent consumed fast food more than three times a week (Francis et al., 2009, p. 1110). As in many other developing countries, eating at a fast food chain, especially a foreign-owned chain, is a status symbol in Jamaica as is the consumption of Coca-Cola and other imported brandname drinks and processed foods.

Numerous studies have tried to assess the link between trade liberalisation and the prevalence of malnutrition, overweight, and obesity. A recent literature review of quantitative studies has concluded that in low-income and middle-income countries trade openness has reduced malnutrition most likely because trade policies enhanced food security and provided a cushion against the impact of international food price rises on the domestic price of staple foods. Moreover it did not discover a positive link between trade openness and overnutrition, possibly because the sheer availability of food is not enough to change consumption patterns. It did, however, find a positive correlation between such globalisation processes as increased TV ownership and internet access and overnutrition (Cuevas García-Dorado et al., 2019). Foreign Direct Investment in Jamaica has been particularly pronounced in the telecommunications sector, allowing for the wide advertising of mostly imported unhealthy foods. From the 1990s onwards, cable TV made its inroads in Jamaica, exporting US food culture and lifestyle. A recent study of the TV watching and food habits of 330 mother-and-daughter pairs in Kingston and St. Andrews concluded that the more Americanised the girls— describing themselves as Jamaican and part-American—the higher their daily consumption of US cable TV and unhealthy food (Ferguson et al., 2018).

The millennium development goals (MDGs) and their successor the sustainable development goals (SDGs) have provided the GoJ with an impetus to address both conventionally understood malnutrition and rising rates of childhood obesity. The SDGs, for instance, include the aim to ‘eradicate *all forms* of malnutrition’. Some of the action undertaken to date to meet this global target include: a national infant and young child feeding policy that aims to increase exclusive breastfeeding for the first 6 weeks; a new poor relief programme in addition to PATH; measures to enhance the knowledge of small farmers; a new food and nutrition policy that aims to make the country more self-sufficient in food production; the formation of a National Food Industry Task Force; and a ban on certain types of sugary drinks in and around schools (Planning Institute of Jamaica, 2018; Ministry of Agriculture and Fisheries and Ministry of Health, 2013; Ministry of Health, 2014; Gleaner, 17 October 2017 and 6 June 2018). Given Jamaica’s increasing incorporation into new economic structures after the 1970s, however, the capacity for such policies to address the double burden of malnutrition will undoubtedly depend on the fortunes of global capital, the priorities of international lenders, and on broader cultural trends into the 2020s.

## Conclusion: towards healthy publics?

The foregoing has shown that child malnutrition in Jamaica gradually declined after independence but that there remained pockets of high child malnutrition, particularly in the poorer rural areas. The overall decline in child malnutrition rates after independence was the result of various factors: an expansion of the primary health care system from the late 1960s onwards, making it easier for mothers to access child welfare and nutrition clinics; the vernacularisation of nutrition education; the adoption of pro-poor policies, ranging from food subsidies and land leasing schemes to food stamps and PATH; and loans and food aid from international aid agencies and lenders. This integration into the global economy, however, also underpinned Jamaica’s rising rates of overweight and obesity towards the end of the 20th century. The resulting exposure to global economic downturns and to international institutions that demanded fiscal austerity worsened the food security of poorer households.

The approach to child malnutrition in post-independence Jamaica shifted from a medicalised approach, focussing on treatment of severe cases, to a more holistic approach that combined treatment with prevention, and which paid attention to cultural beliefs and values, involved multiple sectors, and used lay health workers. Nonetheless, it was very much what Hinchliffe et al. (2018) have called a ‘traditional deficit-led approach’, with a public ‘waiting to be informed or incentivised on an issue’ and interventions based on a predefined and top-down notion on what constitutes as healthy. Breastfeeding campaigns in the 1990s, for instance, were based on WHO guidelines but did not include consultation with the target audience. As many Jamaican women had to work and there were no breastfeeding stations at their place of work, it is not surprising that targets for exclusive breastfeeding were not met.

Hinchliffe et al. (2018, p. 6) have proposed a shift away from a top-down imposition of what it means to be healthy towards the creation of ‘healthy publics’ that bring together specialist and lay groups with different ideas and practices, to ‘co-identify questions and approaches that are relevant to all those affected by the issues; and to generate appropriate forms of evidence that can allow this health and wellbeing knowledge to circulate, gain traction, and contribute towards effective health-care practices and policies’. Such an approach holds out greater promises to address the increasing double burden of child malnutrition in Jamaica than the traditional deficit-led approach, as this problem is far more complex than conventionally understood child malnutrition. It is caused by a multitude of factors that intersect in complex ways and to solve it requires the cooperation of a vast number of institutions, organisations, and interest groups both locally and globally.

There are some signs that the GoJ realises that traditional approaches to nutrition are ineffective to address the double burden of child malnutrition. For example, recognising the contribution of corporations to the problems of childhood obesity, it is increasingly involving the private sector in initiatives to address these issues. For instance, the creation of a National Food Industry Task Force in 2017 brought together stakeholders from the food industry and various other interests groups, including the National Consumers League, the Jamaican Agricultural Society, PAHO and the University of Technology, to develop new dietary guidelines. Also the participation of various Jamaican charities and NGOs in the Healthy Caribbean Coalition’s programme to prevent childhood obesity signals a step away from a traditional deficit-led approach.

Yet it is questionable how easy it is for Caribbean SIDSs, such as Jamaica to create ‘healthy publics’ to tackle the double burden of child malnutrition. First of all, these small economies, heavily reliant on external markets for both exports and imports, are extremely vulnerable to external shocks that can significantly affect the nutritional status of their populations. Take, for example, the Covid-19 pandemic which will reduce tourism earnings, the key economic driver and foreign exchange earner in Jamaica, limiting the country’s ability to import food. The pandemic will also increase unemployment so that more families will struggle to purchase food. Furthermore, children from low-income households will miss out on nutritious school meals because of school closures. And the crisis will also drive up food prices as a result of increased demand for food, changes in the supply chain, etc. (Ewing-Chow, 2020).

And second, Jamaica and most other Caribbean SIDS are patronage democracies; that is, democracies in which ‘citizens, especially those in lower-income groups, are integrated into politics through clientelistic relationships with their political parties’ (Bertelsmann Stiftung, 2018, p. 5). For ‘healthy publics’ to emerge in these countries, there needs to be a shift towards ‘an inclusive, democratic governance process that would stretch the narrow representative nature of the parliamentary system towards a more participatory arrangement’ (Bertelsmann Stiftung, 2018, p. 3). Although Jamaica and other Caribbean SIDS have witnessed the emergence of multi-sectoral civil society coalitions in recent years, such as the Healthy Caribbean Coalition, civil society is at present too weak to put pressure on political leaders and powerful business interests have more influence in policy formulation and agenda setting than civil society organisations.

## Figures and Tables

**Table 1:** Incidence of breast, bottle and combined feeding methods at various ages as percentage of total.

Age	Breast only	Mostly breast	Mostly bottle	Bottle only	No milk
Birth	67	28		5	
6 weeks	23	47	21	10	
3 months	18	27	33	22	
4 months	10	22	41	28	
5 months	6	16	34	44	0.3
6 months	4	14	31	48	2
8 months	4	9	21	63	4
10 months	2	4	17	73	5
1 year	1	2	8	87	3

*Notes*: Date sourced from Grantham-McGregor and Back (1970, p. 405). Their study examined 300 children born at the university hospital between 1967 and 1968.

**Table 2:** Nutritional status of children aged 0–4, 1970–1978.

Gomez scale	1970	1978
Normal	50.1	61.1
Mild	39.1	31.0
Moderate	9.4	3.3
Severe	1.4	0.9

*Note*: Data sourced from Edwards (1984, p. 70).

**Table 3:** Percentage of children malnourished (less than 80 per cent of Harvard standard) by age and year.

Age	1970	1978	1985
0–11 months	13.3	13.5	5.8
12–23 months	25.9	16.7	8.1
24–35 months	17.9	16.0	8.3

*Note*: Data sourced from Sinha (1988, p. 184).

**Table 4:** Type of lunch consumed by school children.

Type of lunch	Percentage
Nothing	6.6
Drink only	6.2
Snack/sweet	27.8
Nutribun	9.9
Patty/sandwich	14.9
School meal	9.8
Meal taken from home	2.8
Meal from shop/vendor	4.2
Went home for lunch	17.7

*Note*: Data sourced from Walker et al. (1998, p. 46).

**Table 5:** Percentage of children under 5, underweight and overweight, 1991–2014.

Year	Underweight	Overweight
1991	5.6	2.3
1992	5.7	3.1
1993	6.1	4.6
1994	4.6	6.2
1995	4.8	5.9
1996	5.6	3.8
1997	3.5	5.5
1998	3.8	4.3
1999	2.1	6.3
2000	3.9	7.3
2001	4.0	5.8
2002	2.7	8.5
2004	3.1	7.5
2006	4.8	6.1
2007	1.8	8.7
2008	2.4	7.7
2010	3.4	4.1
2012	2.7	7.8
2014	2.2	8.3

*Note:* Data sourced from WHO (http://apps.who.int/nutgrowthdb/database/search/Dataset/Search).

## Data Availability

The datasets used in this study were derived from the following public domain resources: https://data.worldbank.org/country/jamaica; http://www.fao.org/faostat/en/#data/FS.

## Notes

1 Most Ministry of Health papers relating to child malnutrition after 1962 have not yet been deposited in the Jamaica Archives. It has not been possible to access the Ministry’s library nor undertake interviews with key public health officials involved in child malnutrition programmes.

2 The Gomez scale is based on a percentage of expected weight for age: over 90 per cent is normal, 76–90 per cent indicates mild (first degree) malnutrition, 61–75 per cent designates moderate (second degree) malnutrition and <60 per cent marks severe (third degree) malnutrition.

3 The programme had started in 1962 by the Church World Service and followed from a match-funded dried-milk distribution scheme set up by UNICEF in 1955. Due to a change in relevant US legislation, responsibility of the programme had to be given to a government agency. From 1967 onwards, the programme was renamed ‘Food for Freedom’ and was administered by the Central Food Organisation.

4 In the 1960s, of the food groups that provided most of the calories 42 per cent was imported and 72 per cent of the food groups that provided most of the protein were imported (Ashworth and Waterlow, 1974, p. 24, p. 39).

5 USAID also sponsored several other food projects, including a kitchen gardens and small livestock scheme in rural Jamaica and a CSM distribution scheme in West Kingston. The former was quite successful as it not only improved people’s diets but also provided an additional income. The latter was no success because little was done to make CSM acceptable (USAID, 1979b).

6 This was partly due to population growth and increased outmigration of medical staff as cuts in government expenditure also affected the medical services.

7 In fact, since the early 2000 in many parts of the Global South there has been a shift towards community-based management of acute malnutrition model under which community health workers diagnose children with uncomplicated severe acute malnutrition and refer them to an outpatient therapeutic feeding programme at health centres (López-Ejeda et al., 2019).

8 The budget cuts related to the SAPs in the 1980s fell disproportionally on schools and health clinics in rural areas. By the end of the 1980s, the state spent more than twice on debt servicing as on social services (Anderson and Witter, 1994, pp. 14–16).

9 Others voted with their feet and migrated overseas. During the 1980s, some 200,000 or 1/12th of the population migrated, mostly to North America (Weiss, 2005, p. 123).

10 A World Bank review of the food stamps programme in the 1980s concluded that while it did not significantly reduce overall poverty, it did lower the poverty levels of certain groups, especially households with elderly residents and households with children (Ezemenari and Subbbarao, 1999).

11 This included, amongst others, a breastfeeding helpline, the use of breastfeeding promoters at hospitals, and support for mothers of new-borns in the work place. It failed to have significant impact: in 2001 only 47 per cent of all new mothers exclusively breastfed for the first 6 weeks (Ministry of Health, 2014).

12 Between 1990 and 1992, a programme funded by the GoJ and UNICEF was trialled at Freeman’s Hall (Trelawny), which used community health volunteers. Contrary to CHAs, these were unpaid positions. Although the annual costs per child per year were modest, the scheme was not rolled out across the island (Melville et al., 1995).
